# Comparison of Different Crosslinking Methods for Preparation of Docetaxel-loaded Albumin Nanoparticles

**Published:** 2015

**Authors:** Hassan Niknejad, Raziyeh Mahmoudzadeh

**Affiliations:** a *Department of Tissue Engineering, School of Advanced Technologies in Medicine, Shahid Beheshti University of Medical Sciences, Tehran, Iran.*; b *Nanomedicine and Tissue Engineering Research Center, Shahid Beheshti University of Medical Sciences, Tehran, Iran.*

**Keywords:** Nanoparticles, Albumin, Cross-linking, Docetaxel

## Abstract

In the last step of desolvation method for preparation of albumin nanoparticles, glutaraldehyde (GA) is added to stabilize the newly formed nanoparticles. Due to undesirable effects of GA, the objective of this study was to evaluate alternative methods of crosslinking including ultraviolet (UV) irradiation, adding of glucose and combination of both methods.

The nanoparticles were prepared by desolvation procedure. Final particle size, zeta potential, FTIR, scanning electron micrograph, cellular uptake and cell toxicity of nanoparticles crosslinked with UV and/or glucose were compared with commonly crosslinked nanoparticles with GA. Moreover, drug release and stability parameters of docetaxel-loaded albumin nanoparticles were investigated.

Size of all nanoparticles prepared by different methods was in the same range (100-200 nm). Zeta potential showed the same results except for those treated with UV. The results of FTIR assay were the same for all groups. Although crosslinking by UV or glucose alone resulted in cytotoxic effects, combination of UV and glucose had less cytotoxic effects compared to GA. Cellular uptake of nanoparticles crosslinked with UV + glucose and GA showed similar results. The release of docetaxel from UV + glucose and GA crosslinked nanoparticles showed the same biphasic release.

These data support the idea that crosslinking with a combination of UV and glucose can be a promising alternative method for production of docetaxel-loaded albumin nanoparticles with the advantage of omitting toxic GA.

## Introduction

A lot of efforts are going ahead to develop drug carrier systems to improve drug performance. In order to achieve this goal, different type of systems are under investigation including nanoparticles (NPs) (1), liposomes (2), dendrimers (3), micelles (4), carbon nanotube (5), molecular conjugates (6) and quantum dots (7). Among these delivery systems, nanoparticles seem to be better choices because they may protect therapeutic agents from degradation and mediate enhanced cell entry. Moreover, nanoparticles may enable the administration of poorly water-soluble drugs. In comparison to the other types of carriers, nanoparticles may allow enhanced target delivery to the desired tissues by different possible surface modifications (8). 

Nanoparticles can be prepared from different synthetic or natural macro-molecular materials. Among all different possible materials, natural nanoparticles made of serum albumin offer several specific advantages (9). Albumin is the most abundant plasma protein (35–50 g/L human serum) which has been used as a drug carrier in several studies. Albumin based particles will be well tolerated without any serious side-effects, which is supported by clinical studies with registered albumin based particle formulations such as Albunex^TM^ (10) and Abraxane^TM^ (11). These nanoparticles have advantage of biodegradability and lack of toxicity and immunogenicity that make them ideal candidates for drug delivery. The body distribution of this drug carrier system is mainly influenced by two physicochemical properties; particle size and surface characteristics (12). Achieving the desired particle size for albumin nanoparticles is easy and reproducible. Moreover, the albumin nanoparticle molecules possess certain functional groups that are available on the particle surface for the covalent attachment of drugs or of drug targeting ligands (13). These groups offer several target sites for covalent modifications such as the Ɛ-amino groups of lysine, the carboxyl groups of aspartic and glutamic acid as well as the hydroxyl groups of tyrosine (9). 

So far, three different methods for albumin-based nanoparticles preparation have been described which include emulsion formation, desolvation and coacervation. Albumin nanoparticles are usually prepared by well-defined desolvation method in which the particles are prepared using the dropwise addition of a desolvating agent (ethanol or acetone) to an aqueous albumin solution at pH between 7 and 9, followed by glutaraldehyde (GA) crosslinking (14,15). GA is generally used for crosslinking and stabilization of albumin derived nanoparticles (16). Crosslinking is an essential step for nanoparticles preparation, which affects biodegradability and drug release from the carrier system (4). However, toxicity of GA and its probable reacting with encapsulated peptide/protein drugs make it an undesirable cross-linker which affects the drugs bioactivity (17). There are some other ways of crosslinking which can be possible alternatives for GA. Physical crosslinking methods including drying, heating, ultraviolet (UV) irradiation and chemical methods including glucose can be other options for crosslinking of nanoparticles (18). All these methods have been previously assayed on other proteins like collagen. It has been shown that ultraviolet irradiation increases tensile strength and affects ultimate percentage elongation of collagen fibers (19). It has been shown that using of UV irradiation and glucose together strengthens glucose-incorporated collagen films (20). However, to the best of our knowledge, there is no report on employing UV and/or glucose as crosslinking agents for preparation of albumin nanoparticles. The aim of this study was to investigate the potential use of UV, glucose and their combination, as possible cross-linkers rather than toxic glutaraldehyde in the production of albumin nanoparticles.

## Experimental

*Chemicals and reagents*

Bovine Albumin (fraction V, purity 96–99%) and glutaraldehyde were obtained from Sigma (Steinheim, Germany). Docetaxel was from Cipla (Mumbai, India). For *in-vitro* cytotoxicity test, amniotic epithelial cells were obtained from elective Cesarean. For cellular uptake, HeLa Cells were obtained from Pasteur Institute. RPMI 1640 and Dulbecco’s Modified Eagle’s Medium (DMEM)/F12 and fetal bovine serum (FBS) were obtained from GIBCO Invitrogen Corporation. 3-(4,5 Dimethylthiazol-2-yl)-2,5-diphenyl tetrazolium bromide (MTT), dimethylsulfoxide (DMSO) and epidermal growth factor (EGF) were from Sigma. 

*Preparation of albumin nanoparticles*

Albumin nanoparticles crosslinked with glutaraldehyde were prepared by desolvation technique as described previously (15). Briefly, 150 mg albumin was dissolved in 2.0 mL of 10 mM NaCl solution, titrated to pH 7-10. The solution was then transformed into nanoparticles by continuous addition of 8.0 mL of the desolvating agent ethanol under stirring (500 rpm) at room temperature. The addition of ethanol was performed by a syringe pump (Model SP-500, JMS Co., LTD; Japan) which enabled nanoparticles preparation at flow rate 1 mL/min of ethanol. After the desolvation process, 8% glutaraldehyde in water was added to induce particle crosslinking (1.175 μL/mg serum albumin). The crosslinking process was performed under stirring of the suspension over a time period of 24 hours. The same preparation technique was used in all other groups, before crosslinking by glutaraldehyde. One group was placed in the UV chamber under UV light (wavelength 254 nm) for 30 minutes. The distance between the light source and the sample was 15 cm (19, 20). The other group was treated with 6 mM glucose (D-glucose, 99.5% anhydrous, Sigma Chemical Co., St. Louis, MO). The same protocol was applied for the third group (UV+ glucose) in which the addition of glucose was followed by immediate exposure to UV source for the same period of time and same intensity and distance, as mentioned above.

*Determination of particle size and zeta potential*

Average particle size and zeta potential were measured by photon correlation spectroscopy (PCS) and micro electrophoresis using a Malvern zetasizer 3000 HSA (Malvern Instruments Ltd., Malvern, UK). The samples were diluted 1:400 with purified water and measured at a temperature of 25 °C and a scattering angle of 90°. Scanning electron microscope showed the shape of albumin nanoparticles after lyophilization.

*Purification of nanoparticles*

The resulting nanoparticles were purified by five cycles of differential centrifugation (15,000 × g, 10 min) and re-dispersion of the pellet to the original volume in 10 mM NaCl at pH 8.2. Each re-dispersion step was performed in an ultrasonication bath (Power Sonic 410, Maihan LabTech, Korea) over 5 min.

*Characterization of* *nanoparticles*

FT-IR spectra were recorded on a 102 MB BOMEM apparatus. Samples for FT-IR measurements were prepared by dissolving them in 1.5 wt% aqueous solution containing phosphate buffer 0.1 M NaCl (pH 7.2). IR spectra were recorded on hydrated film, using AgBr windows with a resolution of 2-4 cm^-1^ and 100-500 scans.

*Cellular uptake of albumin nanoparticles*

HeLa cancer cells were cultured as described previously (21). In brief, the cancer cells were seeded in 24-well-plates at a density of 2×10^4^ cells per well and cultured at 37 °C and 5% CO_2_ in RPMI 1640 (Biochrom, Berlin, Germany) supplemented with antibiotics (100 U/mL penicillin and 100 µ/mL streptomycin; Gibco, Berlin, Germany) and fetal bovine serum (FBS), for about 2 days till the cells reached 70% confluence. Then the cells were incubated with prepared nanoparticles conjugated with fluorescent for 4 hours at 37 °C and 5% CO_2_. For imaging, HeLa cells were gently rinsed with DMEM/FBS twice and subsequently fixed in formaldehyde 4% for 5 minutes. The photos were taken by invert fluorescent microscope. 

*MTT assay*

Human amniotic epithelial (AE) cells were prepared using fresh human placenta after elective Cesarean, as described previously (22). The AE cells were seeded at density of 5×10^4^ per well in gelatin-coated 24-well plate and incubated overnight in incubator (37 °C, 5% CO_2_) in DMEM/F12 supplemented with 10% FBS. The AE cells were incubated with 250 µL suspension of the nanoparticles overnight. Then the cells were washed using phosphate buffered saline (PBS) at pH 7.4. The cells without treatment were used as control group. For cytotoxicity assay, 250 µL of MTT solution (5 mg/mL) was added to each well and the plates were incubated for 4 h. The MTT formazan crystals were then dissolved with 1 mL DMSO at room temperature. The optical density (OD) was measured at 570 nm with a spectrophotometer (CE7500, Cecil, UK). The blank well (containing only medium) was used for zero adjustment. The viable rate was calculated by the following equation:

Viable rate = (OD_treated_/OD_control _)× 100%

 OD_control_ was obtained in the absence of nanoparticles and OD_treated_ was obtained in the presence of nanoparticles.

*Preparation of docetaxel-loaded nanoparticles*

A stock solution of docetaxel (5 mg/mL) was prepared and 200 µL was added to 20 mg of glutaraldehyde and UV + glucose cross-linked albumin nanoparticles. The volume was adjusted with water to 5.0 mL. The mixture was stirred for 4 h (650 rpm) at room temperature to achieve a loading equilibrium of docetaxel.

*HPLC analysis for docetaxel release*

 Dialysis technique was used to evaluate drug release from albumin nanoprticles. A dialysis tube (molecular weight cut-off: 8 kDa) containing prepared docetaxel-loaded nanoparticles (5 mL) was put in a medium consisting of 20 mL PBS (pH =7.4) containing 0.1% Tween 80 and stirred continuously on a shaker incubator for over 12 days at 37 °C. At selected time intervals, 15 mL of the medium was removed and the medium was replenished with freshly prepared PBS. The aliquots were then exposed to dichloromethane for liquid-liquid extraction of docetaxel. The extraction procedure was repeated three times and the extracted organic phase was evaporated and the remnants were dissolved in 200 μL acetonitrile and were subjected to HPLC analysis. Drug release data were expressed as the percentage of the cumulative amount of drug release.

*HPLC analysis of docetaxel*

A Cecil 4200 HPLC system (Cecil instrument Ltd., UK) with UV–Visible detector was used for the quantification of docetaxel in the samples. Mobile phase was an isocratic mixture of water and acetonitrile (70:30) containing 0.1% trifluoroacetic acid and separation was obtained using a reverse phase column. The flow rate was set to 0.8 mL/min and absorption at 230 nm was recorded. The retention time was about 11 min. The levels of docetaxel in the samples were determined from calibration curve.

*Long-time stability of docetaxel-loaded nanoparticles*

Aqueous dispersions of docetaxel-loaded albumin nanoparticles were stored at 4 ºC for a period of 18 months. After pre-determined storage times, the samples were re-dispersed by vortexing for 1 min. As stability parameter, particle size and polydispersity were determined as described above.

*Statistical analysis*

All the quantitative results were expressed as mean ± standard error of the mean (SEM). Statistical analysis was carried out by means of one-way analysis of variance (ANOVA) followed by the Tukey post-test. p-value less than 0.05 were considered statistically significant.

## Results

*Determining of average particle size*

**Figure 1:**
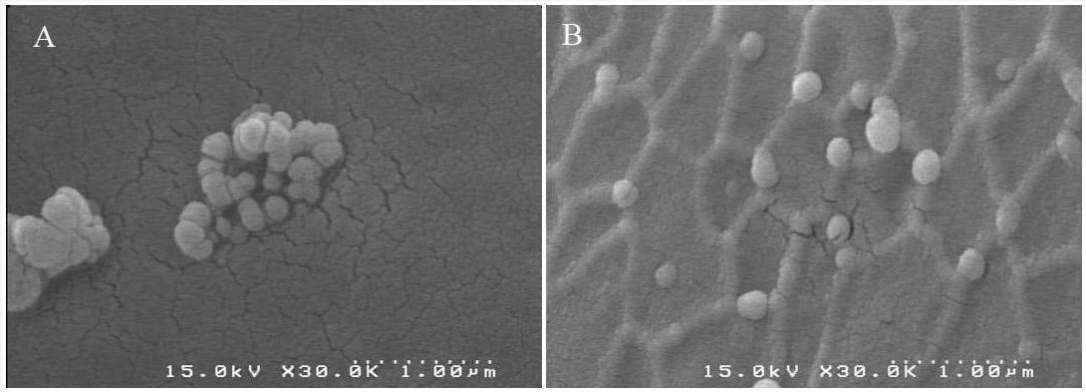
Scanning electron micrographs (SEMs) of albumin nanoparticles prepared by desolvation procedure. At the last step, nanoparticles were crosslinked with GA (A) or UV + glucose (B).

The results of photon correlation spectroscopy (PCS) are shown in Table 1. The nanoparticles prepared and crosslinked with the addition of glutaraldehyde had mean particle diameter of 141.7 ± 22.9 nm with a very narrow distribution (polydispersity index = 0.032 ±0.018). Nanoparticles crosslinked with UV had a size of 134.9 ± 24.6 nm (polydispersity index = 0.030 ± 0.026). Particles which were crosslinked with glucose had mean diameter of 144.6 ± 27.2 nm (polydispersity index = 0.033 ± 0.026). The albumin nanoparticles crosslinked with UV+ glucose showed mean diameter of 140.2 ± 20.1 nm (polydispersity index = 0.034 ± 0.022) (Table 1). No statistically significant differences were observed between 4 groups in diameters and polydispersity index. The SEM micrographs reveal morphological aspects of nanoparticles with a spherical shape and uniform size (Figure 1).

**Table 1:** Mean diameter and zeta potential of albumin nanoparticles prepared with different crosslinking methods. No significant difference was seen in the size of nanoparticles prepared with different crosslinkers. Data are presented as mean diameter ± SEM (Standard Error of Mean) (nm), (n=4) of six independent experiments

**Albumin particles crosslinked by**	**Diameter(nm)** **Mean±SEM**	**Polydispersity index** **Mean±SEM**	**Zeta potential (mV)** **Mean±SEM**
GA	141.7 ± 22.9	0.032 ±0.018	32.5 ± 3.5-
UV	134.9± 24.6	0.030 ± 0.026	-24.9 ± 3.2
GLUCOSE	144.6 ± 27.2	0.033 ± 0.026	-30.1 ± 1.5
GLUCOSE + UV	140.2 ± 20.1	0.034 ± 0.022	-32.4 ± 2.8

*Zeta potential*

There was significant difference between zeta-potential of nanoparticles crosslinked with UV (-24.9 ± 3.2) and nanoparticles crosslinked by glutaraldehyde (-32.5 ± 3.5) (p< 0.01). No statistically significant differences were seen between zeta potentials of particles crosslinked with glutaraldehyde (-32.5 ± 3.5), Glucose (-30.1 ± 1.5) and UV+ glucose (-32.4 ± 2.8) (Table 1).

*FTIR*

**Figure 2:**
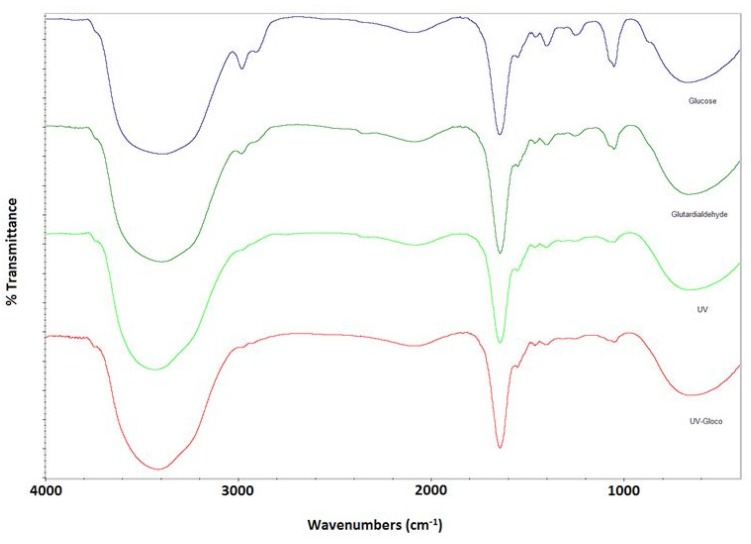
FTIR spectra for nanoparticles prepared with different crosslinking methods.

Figure 2 shows absorption spectra in amides region of albumin. As clearly shown, both Amide II and Amide I spectral contributions are present in all four groups, which is indicative of no significant conformational changes of albumin nanoparticles.

*MTT assay*

**Figure 3:**
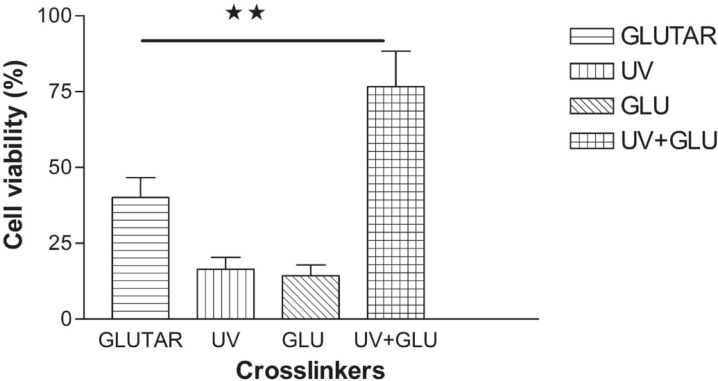
Viability of normal cells treated with nanoparticles which were crosslinked with different methods. The highest level of viability was achieved when the cells were treated with nanoparticles crosslinked with a combination of UV and glucose. Data are presented as Mean ± Standard Error of Mean (n=4) of six independent experiments. (**) P < 0.01

The results in Figure 3 compare toxicity of different groups prepared with conventional and modified methods. Nanoparticles crosslinked with UV and the group crosslinked with glucose showed toxicity for normal cells and significant differences were seen between viability of the cells treated with nanoparticles crosslinked with UV (16.48% ± 3.87) or glucose (14.29% ± 3.60) and nanoparticles crosslinked with glutaraldehyde (40.08% ± 6.6) or combination of UV and glucose (UV + Glucose) (76.59% ± 7.67) (P< 0.01). The cells treated with albumin nanoparticles which were crosslinked with UV + Glucose had the highest level of viability. There was also a significant difference between nanoparticles crosslinked with glutaraldehyde (40.08% ± 6.6) and UV + Glucose group (76.59% ± 7.67) (p < 0.01).

*Cellular uptake of nanoparticles*

**Figure 4:**
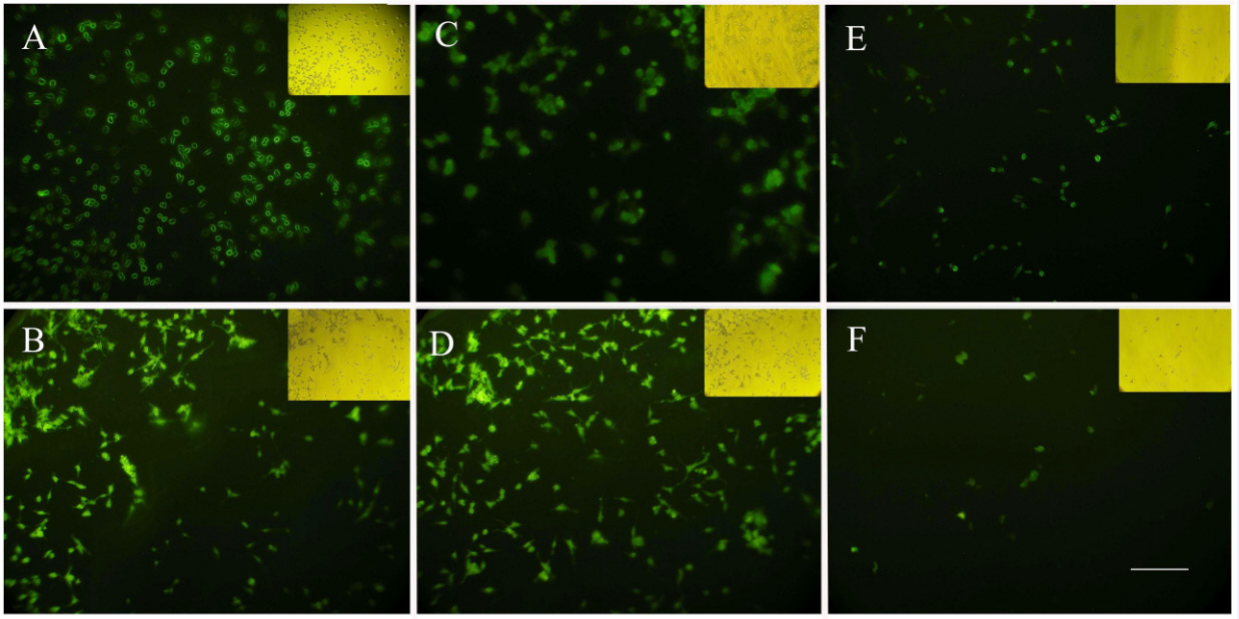
Cellular uptake of albumin nanoparticles prepared with different crosslinking methods. A) GA after 2 h, B) GA after 4 h , C) UV + Glucose after 2 h D) UV + Glucose after 4 h, E) UV (4 h), F) Glucose (4 h). Scale bar: 100 µm

In order to investigate the potential of nanoparticles to serve as a drug carrier, cellular uptake studies were performed with HeLa cells. After 2 h, in the cells incubated with albumin nanoparticles crosslinked by glutaraldehyde, fluorescence was observed solely in the membrane of the cells with decreased signals in the cytoplasm (Figure 4A), while the cells treated with UV + Glucose crosslinked nanoalbumins had enough cytoplasmic uptake after 2 h (Figure 4C). Cytoplasmic accumulation of fluorescence was detected in the cells treated with both UV + Glucose and GA crosslinked nanoparticles after 4 h incubation (Figure 4B and 4D). In the cultures treated with UV- and glucose-crosslinked nanoparticles, after 4 h, the cells showed low cellular uptake which were in consistent with MTT assay data showing a reduced cell viability which may be a reason for reduction of cellular uptake (Figure 4E and 4F).

*Docetaxel release from nanoparticles*

**Figure 5:**
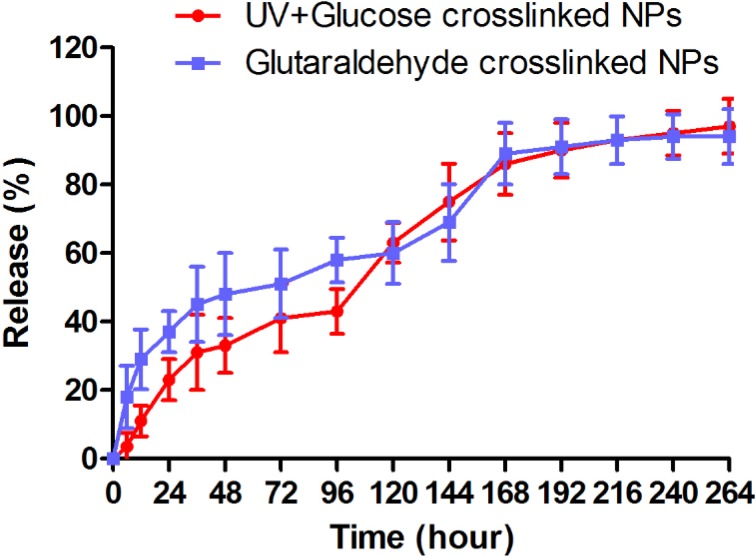
The pattern of docetaxel release from albumin nanoparticles crosslinked with UV+Glucose and glutaraldehyde in PBS medium (pH = 7.4). Both of them showed the same biphasic pattern of docetaxel release

*In-vitro* docetaxel release pattern from nanoparticles crosslinked with glutaraldehyde and UV+Glucose is shown as the cumulative percentage of release in Figure 5. Docetaxel release from both followed a bi-phasic pattern. As shown in Figure 5, a burst release occurred within 24 h when approximately 37% and 24% of docetaxel were released from glutaraldehyde and UV + Glucose crosslinked albumin nanoparticles, respectively. The rest of docetaxel was released from glutaraldehyde and UV + Glucose crosslinked albumin nanoparticles in the same manner with a slower rate within 10 days. 

*Long-time stability of docetaxel-loaded nanoparticles*

**Figure 6:**
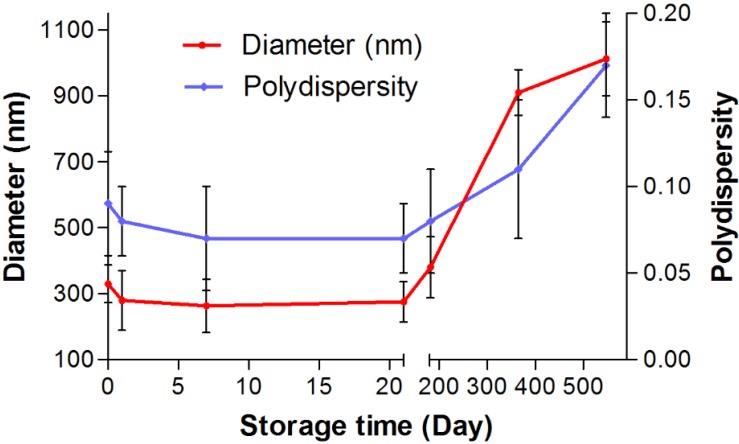
The effect of storage time on particle size and polydispersity of docetaxel loaded albumin nanoparticles cosslinked with UV+Glucose

To consider the storage stability, the docetaxel-loaded nanoparticles crosslinked with UV+Glucose were stored for up to 1.5 years in water at 4 ºC and at predefined times the samples were analyzed with regard to size and polydispersity. After 7 and 21 days of storage, the particles showed a uniform sedimentation (Figure 6). However, the particles were easily re-dispersed by shaking for about 1 min. After 21 days, the particle size slightly decreased (from 330 nm to 274 nm). After 6 months, the nanoparticles still showed an acceptable size 380 nm. There is no significant change in polydispersity after 6 months. After storage periods of 1 and 1.5 years particles became difficult to re-disperse and sizes increased into about the micrometer range with a multimodal size distribution. Therefore, long-term storage of the particles in suspension is not possible. 

## Discussion

The desolvation of albumin with organic solvents followed by chemical crosslinking with glutaraldehyde is a commonly used method for preparation of albumin nanoparticles, which was modified by Langer *et al.* (15). In this method, GA is added at the last step as a crosslinking agent. GA is a colorless liquid with a piquant odor, which is widely used in medicine and industry. It is also used as a fixative in histochemistry and electron microscopy, a cross-linking material and an ingredient of chemical specialty products. However, there are a lot of evidences which indicate toxic effects of GA. Frequently observed effects from exposure to GA include skin sensitivity resulting in dermatitis and irritation of the eyes and nose with accompanying rhinitis (23). In 2006, the cytotoxic and genotoxic effects of glutaraldehyde were studied in different cell lines and in primary cultures of rat hepatocytes and revealed that cytotoxicity of GA include DNA-protein crosslinking and induction of mutations (24). Also in 2005, cytotoxicity of two-component surgical adhesive composed of albumin and GA (BioGlue) used to treat aortic dissections was studied. Based on this study, although GA guarantees strong adherence to tissues and synthetic materials, using GA as a cross-linking agent and its release can result in both *in-vitro* and *in-vivo* cytotoxic effects. Application of glutaraldehyde containing products to lung and liver tissue evoke serious adverse effects such as high-grade inflammation, edema, and toxic necrosis (25). These evidences encouraged us to think about alternative crosslinking methods such as physical methods or other chemical agents. There are several physical crosslinking methods including drying, heating or UV irradiation. Among these methods, UV irradiation seems to be a faster and more effective crosslinking method (26, 27). Moreover, there are other chemical crosslinking agents other than glutaraldehyde such as glucose, which have the same crosslinking properties (28, 29). In this study, the first group was crosslinked with UV exposure and considered for the obtained particles size, zeta potential, FTIR, cell uptake and toxicity for normal cells. These results indicated that UV exposure altered electrical properties of albumin nanoparticles and zeta potential was significantly different with GA group. In addition, toxicity of nanoparticles crosslinked with UV was more than nanoparticles crosslinked with GA. This may be due to toxic free radicals, which are produced from UV irradiation to the nuclei of aromatic residues such as those in tyrosine and phenylalanine (27). Uptake of nanoparticles was influenced by UV crosslinking and approximately 16% of cells were viable after incubation time. Remaining cells that were still alive showed low uptake of nanoparticles. However, size and FTIR of UV cross-linked nanoparticles were similar to other groups and no significant difference was seen. 

Another way of chemical crosslinking was addition of glucose. All the parameters in the nanoparticles crosslinked with glucose, including size, zeta potential and FTIR were similar to GA crosslinked group except for cell toxicity and cellular uptake. The difference in cell toxicity may be due to osmotic changes of medium of the cells after addition of glucose, which resulted in cell death. As discussed previously, cell death can result in reduction of cellular uptake. 

The last group was crosslinked with combination of glucose and GA. The albumin nanoparticles crosslinked with UV + glucose had a desired range of size (100-200 nm). Zeta potential was similar to GA crosslinked group and FTIR study showed no difference. This result is in consistent with previous report achieved in 2007, when a protein based matrix was crosslinked with UV and glucose (28). After addition of glucose, the strength and stability of the matrix were enhanced, which have been ascribed to UV generated free radicals that can expedite crosslinking with glucose via the formation of reactive linear glucose molecules. In other word, once the toxic free radicals are produce by UV irradiation, they immediately contribute to form linear cross-linked complexes with the free glucose molecules. Therefore, “simultaneous” employing of UV and glucose as cross-linker can be a reason for less cellular toxicity; whereas each one alone (in free form) was toxic for the cells. Another study on collagen films revealed that the combination of glucose and UV synergistically improved the mechanical properties and enzyme resistance of collagen films, indicative of increased crosslinking without significant denaturation effects, so that the addition of thiourea (a potent free-radical scavenger) or aminoguanidine (an inhibitor of glucose-derived crosslinking) to the collagen films markedly hindered these synergistic effects (28, 29). The same mechanism probably occurs in albumin nanoparticles crosslinking process. Interestingly, UV + glucose crosslinked nanoparticles were less toxic for the cells than nanoparticles crosslinked with GA, which indicates that crosslinking with UV + glucose, is more desirable than GA for crosslinking of albumin nanoparticles. Moreover, there was no significant difference in docetaxel release from albumin nanoparticles crosslinked with UV + glucose and GA and both showed the same bi-phasic pattern of docetaxel release which was in consistent with previous studies (30). As shown in the results, a burst release of docetaxel occurred within 24 h from glutaraldehyde and UV + Glucose crosslinked albumin nanoparticles. This initial burst release may be related to the portion of docetaxel molecules which are adsorbed on nanoparticle’s surface and the diffusion controlled drug dissolution of drug molecules at nanoparticles and medium interface.

The findings of this study support the idea that UV + glucose crosslinking method is a safe and promising alternative way for preparation of albumin nanoparticles; a procedure which results in producing albumin nanoparticles with the same physical characteristics of GA prepared nanoparticles, but lower toxicity for the normal cells.

## Conclusion

Albumin nanoparticles, the proper carriers in drug delivery systems, can be crosslinked with different methods. In commonly used method, glutaraldehyde is used as a cross-linker which has undesirable effects for the body. In this study, the other possible cross-linkers were used and interestingly the group crosslinked with combination of glucose and UV had the same physical characteristics of nanoparticles prepared with glutaraldehyde, with the advantage of more cellular uptake and less toxic effects for the normal cells. Further studies are required to examine *in-vitro* and *in-vivo* behavior of nanoparticles cross-linked with glucose + UV.
